# Correction: Single nucleotide detection using bilayer MoS_2_ nanopores with high efficiency

**DOI:** 10.1039/d1ra90138a

**Published:** 2021-08-23

**Authors:** Payel Sen, Manisha Gupta

**Affiliations:** Department of Chemical and Materials Engineering, University of Alberta Edmonton Canada payel@ualberta.ca; Department of Electrical and Computer Engineering, University of Alberta Edmonton Canada mgupta1@ualberta.ca

## Abstract

Correction for ‘Single nucleotide detection using bilayer MoS_2_ nanopores with high efficiency’ by Payel Sen *et al.*, *RSC Adv.*, 2021, **11**, 6114–6123, DOI: 10.1039/D0RA10222A.

The authors would like to include additional sentences in their *RSC Advances* article to reference the updated ESI. The paragraph beginning on line four in the right hand column on page 6118 should read as follows:

Fig. 3a–h presents truncated single nucleotide peaks obtained for ML and BL MoS_2_ nanopores for a direct comparison of dwell times. The raw, 100-fold upscaled and the data filtered at 20 kHz for all the different nucleotide translocations for ML and BL MoS_2_ nanopores are shown in Fig. S12. The protocol used for the analysis is also described in the ESI†. It is observed that the dwell times are higher for BL as compared to those of the ML MoS_2_ nanopores for all the different nucleotides. Blockade current is plotted as a function of dwell time for 3000 single nucleotide transport events in Fig. 3i. We observe four distinct blockade current regions for the different nucleotides. Thus, we can conclude that both ML and BL MoS_2_ nanopores are capable of detecting single nucleotides. The blockade current for the nucleotide translocation is plotted as histograms to observe their distribution (ESI Fig. S4†). We observe normal distribution for all the nucleotides for both ML and BL nanopores. Thus, the mean blockade current values along with their standard deviations can be obtained.

The Royal Society of Chemistry apologises for these errors and any consequent inconvenience to authors and readers.

## Supplementary Material

